# La gestión de incendios en hospitales: Limitaciones estructurales y soluciones operativas

**DOI:** 10.23938/ASSN.1155

**Published:** 2026-02-17

**Authors:** Juan Carlos Camacho-Vega, Juan Gómez-Salgado, Javier Fagundo-Rivera

**Affiliations:** 1 Departamento de Construcciones Arquitectónicas II Escuela Técnica Superior de Ingeniería de Edificación Universidad de Sevilla Sevilla España; 2 Unidad de Prevención de Riesgos Laborales Hospital Universitario Virgen Macarena Sevilla España; 3 Departamento de Sociología, Trabajo Social y Salud Pública Facultad de Ciencias del Trabajo Universidad de Huelva Huelva España; 4 Programa de Posgrado de Seguridad y Salud Universidad de Especialidades Espíritu Santo Guayaquil Ecuador; 5 Centro Universitario de Enfermería Cruz Roja Universidad de Sevilla Sevilla España

## Sr. Editor

A nivel mundial, los incendios representan una amenaza significativa para la seguridad en los entornos sanitarios, especialmente en los hospitales. Este riesgo se intensificó durante la pandemia de COVID-19 debido al uso masivo de oxígeno en los tratamientos, lo que incrementó el riesgo de ignición y dio lugar a numerosos incidentes trágicos^1^. 

Diversos incendios ocurridos en instituciones europeas y españolas continúan subrayando la importancia de fortalecer las medidas de protección y mejorar la seguridad en estas infraestructuras. Por ejemplo, la Asociación Alemana de Protección contra Incendios (BVFA) recopiló datos de prensa sobre incendios en instalaciones de salud en Alemania, registrando en 2025 19 muertes y 546 heridos, derivados de 233 incendios en residencias de mayores y 116 en hospitales^2^. A su vez, varios incidentes en España pusieron de manifiesto importantes deficiencias en materia de protección y motivaron una revisión de la normativa de seguridad, como los incendios hospitalarios ocurridos en Sevilla, en 1977 en el Hospital Virgen del Rocío y en 1978 en el Hospital Universitario Virgen Macarena y, más recientemente, en 2024 en el Hospital Fundación Jiménez Díaz de Madrid (que obligó a evacuar varios pisos tras un fallo en la climatización)^3^.

Los centros hospitalarios presentan singularidades que afectan a la gestión de emergencias durante un incendio y que pueden dificultar la evacuación de la zona afectada. A modo ilustrativo, existen zonas con alto riesgo de incendio como los almacenes de farmacia, de material sanitario y de productos químicos (donde se suelen almacenar botellas de gases comprimidos u otros productos inflamables), el área de ambulancias o tránsito de vehículos (frecuentemente localizadas en la entrada principal al centro), o la zona de estar, cocina y descanso para el personal durante los turnos y las guardias (que suele albergar calefactores y dispositivos eléctricos que generan calor). También existen otras zonas a las que resulta más complicado acceder para un rescate, como las consultas más lejanas a las salidas de emergencia, los aseos, los ascensores, las áreas de radiología, las áreas de esterilización, o las estancias ubicadas en sótanos o plantas superiores, donde las personas pueden quedar atrapadas.

Por otro lado, en España existen numerosos edificios hospitalarios que fueron construidos antes de que existieran normativas obligatorias de protección contra incendios. La legislación actual está regulada por el Código Técnico de la Edificación (CTE)^4^ que recoge los requisitos más completos y actualizados. No obstante, adaptar edificios anteriores a esta normativa puede resultar complejo, y el cumplimiento de los requisitos actuales solo es obligatorio cuando se realizan reformas profundas para ajustarse a las normas de protección pasiva que afectan al diseño interior y exterior^5^. En otras palabras: si no hay remodelaciones estructurales significativas, estos edificios pueden seguir operando sin cumplir plenamente los estándares vigentes del CTE para seguridad contra incendios. 

En este contexto, los planes de autoprotección se presentan como una herramienta operativa esencial para la gestión de emergencias en hospitales, ya que permiten organizar la respuesta ante incendios considerando las limitaciones estructurales y funcionales de los edificios y la presencia de pacientes no autónomos o dependientes de soporte vital, especialmente en áreas críticas como quirófanos y unidades de cuidados intensivos^6^. Según el RD 393/2007^7^, el plan de autoprotección no tiene como finalidad identificar carencias ni imponer modificaciones en las instalaciones, sino analizar las deficiencias en evacuación y en seguridad activa y pasiva, organizar la respuesta con los recursos disponibles e implementar mejoras, incluso cuando no se realizan reformas estructurales significativas. Por ello, uno de los retos actuales más importantes es garantizar planes de autoprotección operativos, que dependan tanto del cumplimiento de la normativa en la infraestructura como de la adecuada organización, funciones y formación continua del personal, asegurando que las medidas previstas puedan ejecutarse efectivamente^8^. 

El personal que trabaja en el hospital debe estar entrenado en procedimientos de evacuación, ya que su respuesta puede marcar la diferencia^8^. Además, una evacuación hospitalaria eficaz requiere una estructura de mando y coordinación intraorganizacional que integre a profesionales de distintos roles. Esta cohesión es fundamental no solo para garantizar la comunicación, la gestión de recursos y la correcta ejecución de la evacuación, sino también para identificar áreas vulnerables, evaluar daños, controlar multitudes y coordinarse con los servicios externos de manera eficiente^9^. Decisiones críticas como el corte de gases medicinales, electricidad o climatización deben quedar en manos de estos profesionales^6^.

Más allá de las medidas estructurales y organizativas, la concienciación de pacientes y acompañantes desempeña un papel relevante en la prevención de incendios. Informar sobre el uso adecuado de dispositivos eléctricos, la prohibición de fumar y el respeto a las señalizaciones de seguridad contribuye a reducir conductas de riesgo dentro de los entornos hospitalarios. Asimismo, la realización periódica de simulacros de incendio permite evaluar la eficacia de los planes de autoprotección, detectar deficiencias y mejorar la coordinación entre los distintos servicios implicados^6^. La integración de una cultura de seguridad compartida entre profesionales, pacientes y gestores resulta esencial para prevenir incendios, minimizar su impacto y garantizar una respuesta rápida y eficaz^8^.

Finalmente, la recopilación y análisis de datos históricos sobre incidentes permite identificar áreas vulnerables, evaluar la efectividad de las medidas implementadas y ajustar los planes de autoprotección de forma continua. Para ello, el uso de tecnologías avanzadas, como *software* de simulación, realidad virtual e inteligencia artificial, mejoran la eficacia de los planes de autoprotección al analizar la dinámica del fuego, el comportamiento del humo, las rutas de evacuación alternativas y las posibilidades de compartimentación. Además, la integración de sensores de detección temprana, sistemas de alarma automatizados y controles inteligentes de instalaciones críticas (como oxígeno, electricidad y climatización) permiten anticipar riesgos y activar protocolos de respuesta de manera inmediata^10^. 

Así pues, la seguridad contra incendios en hospitales depende de la infraestructura, la organización interna y la formación del personal. Los planes de autoprotección deben ser operativos, adaptarse a cada edificio y evaluarse continuamente. La integración de tecnologías avanzadas y la colaboración entre profesionales y pacientes son clave para anticipar emergencias, optimizar la respuesta y proteger vidas.

## Data Availability

Se encuentran disponibles bajo petición al autor de correspondencia.
